# Predictors and outcome of time to presentation among critically ill paediatric patients at Emergency Department of Muhimbili National Hospital, Dar es Salaam, Tanzania

**DOI:** 10.1186/s12887-022-03503-y

**Published:** 2022-07-22

**Authors:** Alphonce N. Simbila, Said S. Kilindimo, Hendry R. Sawe, Zawadi E. Kalezi, Amne O. Yussuf, Hussein K. Manji, Germana Leyna, Juma A. Mfinanga, Ellen J. Weber

**Affiliations:** 1 Emergency Medicine Department, Muhimbili University of Health and Allied Sciences, P.O. Box 65001, Dar es Salaam, Tanzania; 2 Emergency Medicine Department, Muhimbili National Hospital, Dar es Salaam, Tanzania; 3 Department of Epidemiology and Biostatistics, Muhimbili University of Health and Allied Sciences, Dar es Salaam, Tanzania; 4 Department of Emergency Medicine, University of California, San Francisco, CA USA

**Keywords:** Paediatric patient, Critically ill, Delay, Mortality

## Abstract

**Background:**

Mortality among under-five children in Tanzania remains high. While early presentation for treatment increases likelihood of survival, delays to care are common and factors causing delay to presentation among critically ill children are unknown. In this study delay was defined as presentation to the emergency department of tertially hospital i.e. Muhimbili National Hospital, more than 48 h from the onset of the index illness.

**Methodology:**

This was a prospective cohort study of critically ill children aged 28 days to 14 years attending emergency department at Muhimbili National Hospital in Tanzania from September 2019 to January 2020. We documented demographics, time to ED presentation, ED interventions and 30-day outcome. The primary outcome was the association of delay with mortality and secondary outcomes were predictors of delay among critically ill paediatric patients.

Logistic regression and relative risk were calculated to measure the strength of the predictor and the relationship between delay and mortality respectively.

**Results:**

We enrolled 440 (59.1%) critically ill children, their median age was 12 [IQR = 9–60] months and 63.9% were males**.** The median time to Emergency Department arrival was 3 days [IQR = 1–5] and more than half (56.6%) of critically ill children presented to Emergency Department in > 48 h whereby being an infant, self-referral and belonging to poor family were independent predictors of delay. Infants and those referred from other facilities had 2.4(95% CI 1.4–4.0) and 1.8(95% CI 1.1–2.8) times increased odds of presenting late to the Emergency Department respectively. The overall 30-day in-hospital mortality was 26.5% in which those who presented late were 1.3 more likely to die than those who presented early (RR = 1.3, CI: 0.9–1.9). Majority died > 24 h of Emergency Department arrival (*P*-value = 0.021).

**Conclusion:**

The risk of in-hospital mortality among children who presented to the ED later than 48 h after onset of illness was 1.3 times higher than for children who presented earlier than 48 h. It could be anywhere from 10% lower to 90% higher than the point estimate. However, the effect size was statistically not significant since the confidence interval included the null value Qualitative and time-motion studies are needed to evaluate the care pathway of critically ill pediatric patients to identify preventable delays in care.

## Background

Critical illness is a life-threatening process which, without timely medical or surgical intervention, is highly likely to result in death [1]. Half of the deaths among children under five years globally occurred in sub-Saharan Africa in 2017. Unlike in high income countries where 1 in 185 children died before the age of five years, 1 in 13 died before the age of five in Sub-Saharan Africa. The risk of dying for a child in sub-Saharan Africa is 15 times higher than in Europe. Most of these children die due to treatable and preventable causes such as complications of during birth, pneumonia, diarrhea, malaria and neonatal sepsis [2]. A 2010 survey by the ministry of health in Tanzania showed high mortality rate in under-fives with 75% of deaths occurring in the first 24 to 48 h after admission [3]. The unfolding of events in the process of critical illness is influenced by multiple factors which can potentially modify and affect the outcome of critical illness. In addition to improvements in the quality of care for children reducing delays in critical illness is among the main approaches to reducing mortality.

Paediatric critical illness in the Low-and Middle-Income Countries (LMICs) differs from the developed countries in that children tend to be younger and suffer more from infectious causes of illnesses. Late presentations to the hospital due to referrals and travels through long distances to reach hospitals are common occurrence which contribute to an increased disease severity and mortality upon admission [4].

Delayed presentation to the hospital in the course of paediatric critical illness has been shown to be one of the factors that negatively influence health outcomes [5]. Delayed health care seeking of more than 48 h has been observed in 35% of paediatric patients in the acute phase of illness in Kigali, Rwanda [6]. In Southwestern Uganda, 50% of paediatric patients who presented late to the health facility and were admitted died within 24 h of admission [7]. At a tertiary hospital in Ethiopia about 4. 1% of children died at the Paediatric Emergency Department, which translates to a mortality of 8.2 per 1000 children. Delay of more than 48 h since the onset of symptoms was among the top causes of early mortality [5]. In Dodoma Tanzania, the median time of delay to seek care among children under five years with fever was 2 days [8].There is an association between delayed Intensive Care Unit (ICU) admission and mortality. Fraction of mortality attributable to ICU delay was 30% [9]. Many studies in this area have looked at timeliness at the level of the family which affects the first phase in the Thaddeus and Maine’s delay model [10].

Presence of a full capacity ED, Paediatric ICU and definitive paediatric services at MNH (Muhimbili National Hospital) have provided early resuscitation, stabilization and other management for critically ill paediatric patients. The objective of this study was to describe patterns, determine factors associated with delay and find out whether delay made a difference in mortality outcome among critically ill children who sought care at the ED of a national tertiary referral hospital.

## Methods

### Study design

This was a prospective observational cohort study of paediatric patients aged 28 days to 14 years triaged ESI level 1 triage presenting to the MNH ED from September 2019 to January 2020.

### Study setting

The study was conducted at ED of MNH which is a national tertiary referral government hospital located in Dar es Salaam with 1500 bed capacity. The MNH serves an annual average of 60,000 patients who are referred from all over the country. The ED at MNH is full capacity public ED in Tanzania and the only training site for Emergency Medicine residency program in the country. Daily the MNH ED attends about 150–200 critically ill patients, among them approximately 25% are children excluding neonates who present directly to the maternity unit. The MNH ED uses a triage system modelled on the ESI but with only 3 levels, with emergent being equivalent to levels 1 and 2 of ESI, and they are assigned to the resuscitation room. Apart from ability to provide full resuscitation including continuous cardiopulmonary monitoring, the department also has a full range of point of care tests like ultrasound, chemistry, blood gas analyzer and portable x-ray. After resuscitation and stabilization, the critically ill children get admitted to general pediatric ward or pediatric intensive care unit (PICU).

### Study participants

We included all pediatric patients (aged 28 days to 14 years) triaged as emergent whose parents/guardians consented to participate in the study.

### Study protocol

Consecutive sampling technique was employed to enroll patient s whereby data collected for 24 h/day on alternate days. Demographics, clinical presentation, initial management, and ED outcomes were observed and documented using information given by the parent/guardian, the treating physician, and the electronic medical record (Wellsoft™) using structured case report form. All patients were asynchronously followed up in hospital wards (if admitted) to determine their in-hospital outcome (discharge/mortality) in 24 h and later weekly for a maximum period of 1 month after admission.

### Measurements

Each caretaker of a critically ill paediatric patient was asked about the date and time of onset of an index illness of the child. Time of presentation to the MNH ED was therefore calculated by finding the difference between these. Delay was defined as presentation to MNH ED after 48 h from onset of illness. Wealth was determined based on household characteristics and asset ownership hence relative household wealth index was constructed using principal component analysis (PCA). Households were then ranked in ascending order. The scores were separated into quintiles; each representing 20% of the population. Those in the highest quintile might not have been rich but were in higher socioeconomic status than 80% of the participants in this study.

### Outcomes

The primary outcome was the association of delay with mortality and secondary outcomes was predictors of delay among critically ill paediatric patients.

### Data analysis

Data were imported into the Statistical Package for Social Science for analysis (SPSS) (version 26.0, IBM, LTD, North Carolina, USA) from the Research Electronic Data Capture (RedCap version 7.2.2, Vanderbilt, Nashville, TN, USA). Relevant frequencies and tables were generated for categorical variables (injury and referral factors). Medians/inter-quartile ranges were calculated for continuous variables. We calculated a proportion of children with delayed presentation and contingency tables were constructed for univariate analysis to explore differences between children who had a delayed vs. timely presentation using the Chi-square test. Multivariate logistic regression analysis was completed on variables with *p* value ≤ 0.20 in the univariate analysis to identify independent predictors of delayed presentation. Relative risks were computed for association of delay with overall, early and late mortality. The odd ratios and 95% confidence intervals were estimated for each studied factor. Statistical significance was set at *p*-value < 0.05.

## Results

**Fig. 1:**
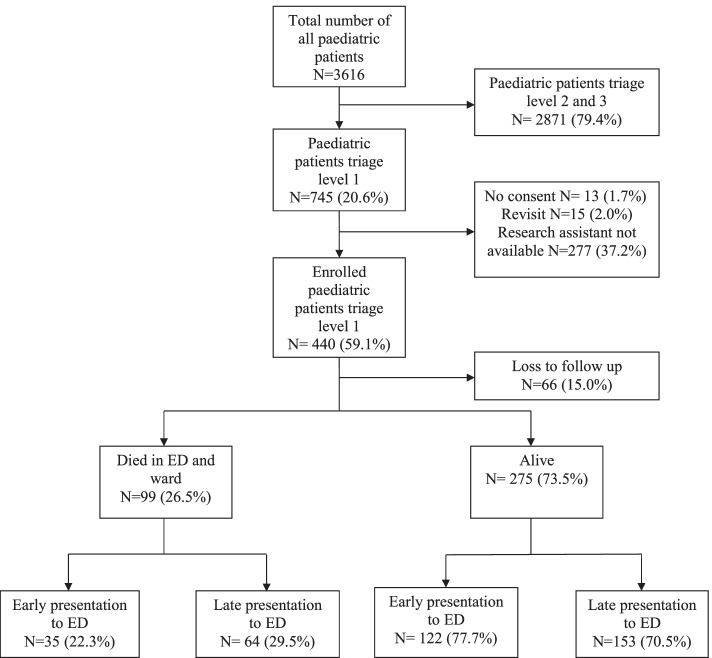
Flow chart of paediatric patients

A total of 3616 paediatric patients attended the ED during the study period, of whom 745 (20.6%) were triaged “emergent”. A total of 440 (59.1%) patients were eligible and consented to participate in the study. Of the 99(26.5%) who died within 30 days of presentation, 64(64.6%) presented late (after 48 h) and 35 (35.4%) presented early (before 48 h) (Fig. 1).

### Socio-demographic characteristics of critically ill paediatric patients at the MNH ED and their caretakers

In the studied cohort, majority 281(63.9%) were males, the median age was 12 [IQR = 9–60] months and majority 321(73.0%) were referred from a lower-level health facility. Most of the caretakers of the critically ill paediatric patients were parents 410(93.2%) and more than half of them 260(59.1%) were between the age of 25 and 34 years. Slightly more than half of them 236(53.6%) had primary education, 145(33.0%) were unemployed, 46(10.5%) were peasants and 59 (67.8%) were the poorest in the socioeconomic status. (Table 1) Of the paediatric patients, 72% were admitted to the ward, 19% were admitted to the PICU and 9% (33/374) died while receiving care at the ED.

**Table 1:** Socio-demographic characteristics of paediatric patients with ESI triage level 1 at MNH ED

Variable	Category	Median [IQR]	Frequency (%) *N* = 440
**Age (months)**	< 12		142 (32.3)
	12- < 60		181 (41.1)
	≥ 60		117 (26.6)
	Median [IQR]	12 (9–60)	
**Sex**	Male		281 (63.9)
	Female		159 (36.1)
**Type of referral**	Facility		321 (73.0)
	Self-referral		119 (27.0)
**Caretaker**	Parent		410 (93.2)
	Guardian		30 (6.8)
**Age of caretaker (years)**	< 25		47 (10.7)
	25–34		260 (59.1)
	≥ 35		133 (30.2)
	Median [IQR]	32 (28–36.7)	
**Level of education of caretaker**	No formal education		32 (7.3)
	Primary education		236 (53.6)
	Secondary education		141 (32.0)
	University/college		31 (7.0)
**Occupation status of caretaker**	Employed		55 (12.5)
	Self employed		194 (44.1)
	Unemployed		145 (33.0)
	Peasant		46 (10.5)
**Socioeconomic status**	Poorest		87 (19.8)
	Poor		91 (20.7)
	Medium		92 (20.9)
	Rich		79 (18.0)
	Richest		91 (20.7)

### Magnitude and predictors of delay

Among the 440 critically ill children, 249/440 (56.6%) had delayed presentation with median time interval from onset of illness until presenting to the ED – MNH was 3 days with IQR [1–5] (Table 2). After multivariate logistic regression, less 1 year old, referred patients, and poor socioeconomic status were independent predictors of delay presentation with OR 2.4, 1.8 and 2.4 respectively (Table 3).

**Table 2:** Predictors of delayed presentation to MNH ED among paediatric patients with ESI triage level 1

Variable	Timeliness, N (%)		OR (95%CI)	*P*- value
	**Early**	**Late**		
**Age of a child (Months)**	***N* = 191**	***N* = 249**		
< 12	48 (33.8)	94 (66.2)	2.2 (1.3–3.7)	**0.002**
12–60	81 (44.8)	100 (55.2)	1.4 (0.9–2.2)	0.17
≥ 60^a^	62 (53.0)	55 (47.0)		
**Sex**				
Male	131 (46.6)	150 (53.4)	0.7 (0.5–1.0)	**0.07**
Female^a^	60 (37.5)	99 (62.3)		
**Type of referral**				
Self-referral^a^	66(55.5)	53(44.5)		
Facility referral	125(38.9)	196(61.1)	2.0 (1.3–3.0)	**0.002**
**Level of education of a caretaker**				
No formal education^a^	10 (31.3)	22 (68.8)		
Primary education	101 (42.8)	135 (57.2)	0.6 (0.3–1.3)	**0.20**
Secondary and higher education	80 (46.5)	92 (53.5)	0.5 (0.2–1.2)	**0.11**
**Occupation status of a caretaker**				
Employed	30 (54.5)	25 (45.5)	0.7 (0.4–1.2)	**0.16**
Self-employed/ Business^a^	85 (43.8)	109 (56.2)		
Unemployed	76 (39.8)	115 (60.2)	1.2 (0.8–1.8)	0.42
**Socioeconomic status**				
Poorest	28 (32.2)	59 (67.8)	2.4 (1.3–4.3)	**0.006**
Poor	40 (44.0)	51 (56.0)	1.4 (0.8–2.6)	0.24
Medium	41 (44.6)	51 (55.4)	1.4 (0.8–2.5)	0.27
Rich	34 (43.0)	45 (57.0)	1.5 (0.8–2.7)	0.21
Richest^a^	48 (52.7)	43 (47.3)		

^a^**Reference**

**Table 3:** Multivariate analysis of predictors of delayed presentation to MNH ED among paediatric

**Variable**	OR (95%CI)
**Age (Months)**	
< 12	**2.4 (1.4–4.0)**
12–60	1.5 (0.9–2.5)
≥ 60^a^	
**Sex**	
Male	0.7 (0.5–1.0)
Female^a^	
**Facility referral**	
Self-referral^a^	
Facility referral	**1.8 (1.1–2.8)**
**Level of education of a caretaker**	
No formal education^a^	
Primary education	0.7 (0.3–1.6)
Secondary and higher education	0.9 (0.3–2.2)
**Occupation status of a caretaker**	
Employed	1.0 (0.7–1.6)
Self-employed/ Business^a^	
Unemployed	0.7 (0.4–1.4)
**Socioeconomic status**	
Poorest	**2.4 (1.2–4.8)**
Poor	1.4 (0.8–2.7)
Medium	1.7 (0.9–3.1)
Rich	1.5 (0.8–2.8)
Richest^a^	

^a^**Reference**

### Mortality and delay among critically ill paediatric patients

**Fig. 2:**
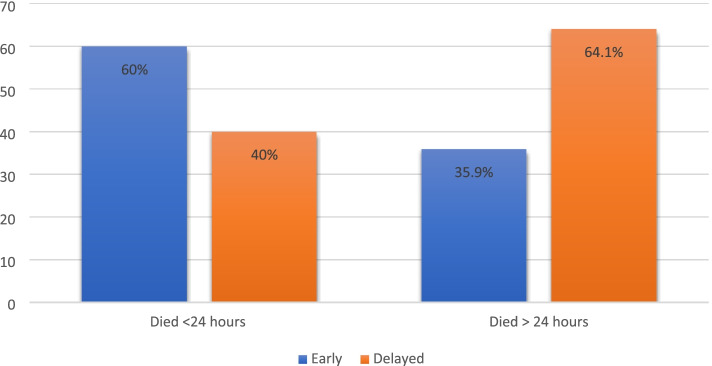
Distribution of early and late mortality vs early and late presentation among paediatric patients with ESI triage level 1. *(*p*-value = 0.021)

Of all critically ill patient, 374 (85%) completed follow up, among them 44(11.8%) died within 24 h which makes total of 99(26.5%) died within 30 days. Of those who died, 64(64.6%) presented late to the ED which were 1.3 times more likely to die compared to those who came early. (RR 1.3 (95% CI: 0.9–1.9) (Table 4). However, among those who died after 24 h a higher proportion (64.1%) had delayed presentation (*p*-value = 0.021) (Fig. 2).

**Table 4:** Association of delay with overall mortality among paediatric patients with ESI triage level 1

Variable	Mortality N (%)		Relative Risk (95% CI)
	Dead (*N* = 99)	Alive (*N* = 275)	
Delayed presentation	64 (29.5)	153(70.5)	1.3 (0.9–1.9)
Early presentation	35 (22.3)	122 (77.7)

## Discussion

This prospective cohort study aimed to determine factors associated with delay to presentation among critically ill children who seek care at a tertiary referral hospital.

Delay to seek care has been reported in several studies as a cause of avoidable morbidity and mortality in children. In our study over fifty percent of critically ill paediatric patients presented late (after 48 h from onset of illness) to the ED. This is similar to findings by a study in Ethiopia but higher than that observed in Rwanda with delay of thirty five percent of paediatric patients [5, 6]. A possible explanation could be differences in sociodemographic characteristics and lifestyle of the study settings. The hierarchical referral system in the setting of this study could contribute to the observed delay as this study was conducted in tertiary hospital which is the highest destination in the chain of referral [11].

In this cohort almost a quarter of the critically ill children who presented to the ED after 48 h from onset of illness died with almost two- thirds of the deaths occurring beyond 24 h of hospital admission. This is similar to findings by a study done at a tertiary hospital in Ethiopia [5]. This may signify the impact of resuscitation and stabilization at the ED before admission that presumably prolonged their lives beyond 24 h but later decompensated [12]. However, limited number of beds in paediatric ICU could explain the occurrence of death beyond ED stabilization. Scarcity of beds in the paediatric ICU deterred doctors at the emergency department from admitting critically ill paediatric patients to the ICU. They instead admitted them to general wards which had no intensive care capacity.

We also found that critically ill children below one year of age were more likely to present late to the ED. Non-specific symptoms especially in infants and caretakers’ poor knowledge on danger signs of critical illnesses might contribute to delay in this age group [13]. Our study also found coming from the poorest households independently doubled the odds of a critically ill paediatric patient being late to the tertiary hospital during a critical illness. This is in keeping with a study conducted in Ethiopia which had similar findings [14].

About two thirds of the critically ill paediatric patients who delayed were referred from primary health care facilities with no capability to care for critically ill children. Failure to recognize critical illness with series of evaluations by primary health care providers before they are referred to definitive care significantly lead to delay with limited management [15]. The existing referral system in place [11] doesn’t take into account the urgency to definitive care hence more delay with poor outcome observe.

## Limitations

This study was conducted in urban settings and single center tertiary hospital with full capacity to resuscitate and stabilize critically ill paediatric patients. However, the MNH ED receives referrals from all over the country, the patients sampled are likely to provide a true representation of the Tanzanian population of critically ill paediatric patients.

Patients were only enrolled when researchers were in the department; some of potential participants might have been missed. However, as the researchers worked different shifts, there is no reason to expect that the missed patients were different from those that were enrolled.

There was loss to follow up encountered in this study. However, it was mitigated by including 10% loss to follow up rate during the estimation of the sample size of the study participants who were to be included during the development of the study protocol.

## Conclusion

The proportion of delayed presentation to tertiary hospital among critically ill paediatric patients is substantially high. The risk of in-hospital mortality among children who presented to the ED later than 48 h after onset of illness was 1.3 times higher than for children who presented earlier than 48 h. It could be anywhere from 10% lower to 90% higher than the point estimate. However, the effect size was statistically not significant since the confidence interval included the null value. Age below one year, being referred from a primary healthcare facility and being in the poorest category of socioeconomic status predicted delay to appropriate care facility. Qualitative and time-motion studies are needed to evaluate the care pathway of critically ill paediatric patients to identify preventable delays in care.

## Data Availability

The dataset supporting the conclusion of this article is available from the authors on request.
